# Understanding participants’ experiences of a behaviour change intervention within cardiac rehabilitation: A nested process evaluation within the STRENGTH randomised controlled trial

**DOI:** 10.1371/journal.pone.0351117

**Published:** 2026-06-16

**Authors:** Clare T. M. Doherty, Mark A. Tully, Jason J. Wilson, Victoria Irving, Lisa Spratt, Rachel O’Reilly, Kim Kensett, Nicole E. Blackburn

**Affiliations:** 1 School of Health Sciences, Ulster University, Derry-Londonderry, United Kingdom; 2 School of Medicine, Ulster University, Derry-Londonderry, United Kingdom; 3 School of Sport and Exercise Science, Ulster University, Derry-Londonderry, United Kingdom; 4 Better Belfast, Greenwich Leisure Limited, Belfast, United Kingdom; 5 National Health Service, Belfast Health and Social Care Trust, Belfast, United Kingdom; 6 South Eastern Health and Social Care Trust, Lisburn, United Kingdom; 7 Belfast Health Development Unit, Public Health Agency, Belfast, United Kingdom; University Hospital Cologne: Uniklinik Koln, GERMANY

## Abstract

**Background:**

The STRENGTH (Self-management and Theory-based Rehabilitation Encouraging New Gateways to Healthy-Hearts) study evaluated a behaviour change intervention embedded within cardiac rehabilitation for individuals with coronary heart disease. This manuscript reports the process evaluation, which aimed to explore participants’ experiences of physical activity, barriers and facilitators to maintaining physical activity, and contextual and process-related factors influencing engagement with the intervention.

**Methods:**

A process evaluation was nested within a two-arm cluster randomised controlled trial. Semi-structured interviews were conducted with participants from both intervention and control groups, focus groups were conducted with intervention participants. Thematic analysis examined factors influencing physical activity maintenance, including context, implementation, mechanisms of impact and perceived outcomes. Quantitative procedures measured intervention fidelity through attendance records and participant diaries.

**Results:**

Both groups highlighted environmental, social, and personal factors influencing participation in cardiac rehabilitation, such as social support, professional oversight, and structured programming. Trainers and peer interactions were valued for motivation and perceived safety. The intervention group reported additional facilitators, including tailored support, external accountability, and self-monitoring, which increased awareness and reinforced adherence. Mechanisms of impact included increased awareness of physical activity, greater exercise confidence, and a sense of accomplishment. A perceived lack of exercise confidence may have hindered independent physical activity engagement.

**Conclusions:**

While behaviour change strategies can enhance engagement in cardiac rehabilitation, their impact on measurable health outcomes remains unclear. Social support, professional oversight, and structured programming appear effective in maintaining physical activity levels, but future interventions should prioritise exercise confidence and education to promote sustained behaviour change.

**Trial registration:**

Registered with ClinicalTrials.gov on 26-01-2023 (ID: NCT05705310).

## Background

Cardiovascular diseases (CVD), including coronary heart disease (CHD), are primary causes of death and disability globally [1,2]. Although cardiac rehabilitation (CR) reduces mortality, morbidity, unplanned hospital admissions and improves quality of life and psychological well-being [3], sustaining physical activity (PA) after formal CR remains a major challenge. Adults who engage in regular PA have lower rates of many serious health conditions including, not only CHD, but also depression, type II diabetes mellitus, hypertension, multiple cancers, stroke, and even premature death [4]. Indeed, PA, which is a major component of CR in the UK, has been shown to be as effective as some commonly prescribed medications (such as statins, β-blockers and angiotensin-converting enzyme inhibitors) at combating early mortality [5]. For adults, the recommended level of PA is at least 150 minutes of moderate physical activity or 75 minutes of vigorous PA weekly, or a combination of both moderate-vigorous physical activity (MVPA) [6,7].

Following completion of structured CR, individuals in the UK are often referred to local exercise programmes. Although participation in CR has been shown to enhance PA levels [8], several studies indicate that these improvements are unlikely to be maintained independently once the formal CR programme ends [9,10]. Walking, a low-impact and accessible form of PA, has been shown to effectively improve fitness and sustain activity levels [11], with pedometer-based interventions proving successful in increasing walking [12–14]. For PA to provide lasting health benefits, it must be practiced in perpetuity [15], highlighting the importance of interventions focused on promoting maintenance.

Interventions that integrate behaviour change strategies, such as enhancing self-efficacy, developing problem-solving skills, and implementing relapse prevention techniques, may help sustain positive lifestyle changes beyond the maintenance stage of CR [16]. Self-efficacy, reflecting an individual’s confidence in their ability to plan and perform behaviours to achieve desired outcomes, is a key target of these interventions [17]. The STRENGTH (Self-management and Theory-based Rehabilitation Encouraging New Gateways to Healthy-Hearts) study was designed to target CHD patients in the maintenance stage of CR with a behaviour change complex intervention embedded into existing services. This emphasis on self-efficacy provides important context for interpreting participants’ reported confidence and links the theoretical basis of the intervention to our qualitative findings.

To evaluate the effectiveness of the behaviour change intervention, the study used both quantitative impact evaluation [18] and mixed methods process evaluation, which we focus on in this paper. The process evaluation included quantitative assessment of fidelity and mediating factors, as well as qualitative exploration of participants’ experiences.

While previous research has demonstrated that cardiac rehabilitation improves physical activity in the short term, maintaining these gains following programme completion remains inconsistent, and the effectiveness of behaviour change strategies in supporting long-term maintenance is variable across studies [8]. Importantly, less is known about how such interventions work in practice within real-world maintenance-stage cardiac rehabilitation settings, particularly regarding the contextual, behavioural, and implementation factors that influence sustained physical activity [19].

Specifically, this process evaluation aimed to examine the context in which the study occurred, the implementation of the intervention, the mechanisms of impact and both measured and perceived outcomes, providing a comprehensive understanding of participants’ engagement and experiences.

## Methods

### Study design

The analysis is based on the STRENGTH study, a cluster randomised controlled trial (RCT) evaluating the effectiveness of a multi-component intervention aiming to promote sustained changes in PA following completion of maintenance stage CR. This process evaluation was conducted within the larger STRENGTH study, with the quantitative findings published separately. The study followed the Consolidated Criteria for Reporting Qualitative Research (COREQ) guidelines [20]. RCTs are commonly used to assess the effectiveness of public health interventions. However, evaluating complex interventions such as behaviour change programmes presents challenges for RCTs [21], due to factors such as the number of components in the experimental and control interventions, the behaviours required from those involved, the number of targeted groups or levels, as well as the added complexity of multiple or non-linear outcomes [22,23]. Such variability in implementation can therefore make interventions difficult to replicate. Moreover, it is often difficult to identify the impact of these interventions using quantitative measures alone.

Qualitative research methods are increasingly being used to gain deeper insights by capturing the lived experiences of participants and exploring contextual factors that influence outcomes [24,25]. These approaches are particularly valuable for understanding interventions that aim to change human behaviour, as they highlight the underlying processes and circumstances that can impact results [26].

In line with MRC Guidance on Process Evaluation [27], this study adopted a mixed-methods framework examining whether the intervention was delivered and received as intended (implementation), how intervention activities, and participants’ interactions with them, impacted behaviour change (mechanisms of impact; through behavioural processes and review of intervention attendance records and participant diaries), how potential external factors may have influenced the delivery and functioning of the intervention (context; through post-intervention focus groups); and which effects could be attributed to the intervention, as perceived by the participants (perceived effects; through post-intervention interviews with both the intervention and control groups). Quantitative procedures measured the fidelity of the intervention through attendance records and participant diaries. They also included the assessment of mediating factors (PA and self-efficacy). Qualitative procedures addressed the same research question, according to participants’ perceptions and researchers’ observations. In order to provide deeper insights and a greater understanding of the impact and effect of the STRENGTH intervention, a range of qualitative methodologies were nested in the trial including individual interviews and focus groups.

### Setting and participants

STRENGTH study participants (n = 96) were recruited into the STRENGTH study from six different CR classes across two counties in Northern Ireland between 6 March 2023 and 16 February 2024 (Control: n = 44, Intervention: n = 52). Participants were 65.04 ± 8.38 (Mean ± standard deviation [SD]) years old and were majority male (75%), see Table 1. Upon recruitment, all participants gave written informed consent that they understood they may be invited to take part in an interview and/or focus group discussion surrounding their experience of the intervention, and that their participation was voluntary.

**Table 1:** Breakdown of descriptive information for full study cohort, and by condition.

	N	Mean Age ± SD	Sex
**All Participants**	96	65.04 ± 8.00	F = 24; M = 71; Unanswered = 1
**Control**	44	63.82 ± 8.74	F = 9; M = 35
**Intervention**	52	66.10 ± 7.99	F = 15; M = 36; Unanswered = 1

Abbreviations: F = female; M = male.

### Intervention

Participants in both arms attended standard maintenance-stage CR, typically comprising two supervised 60-minute exercise sessions per week delivered by leisure-centre exercise professionals trained in CR. Sessions included warm-up, aerobic and resistance exercise, and cool-down, delivered at moderate intensity (Borg RPE 11–14) and tailored to individual functional capacity.

Participants in the intervention arm received the STRENGTH behaviour change programme alongside standard CR. The intervention was informed by behaviour change theory and designed to enhance self-efficacy and self-regulation for PA maintenance. It incorporated established behaviour change techniques, including goal-setting, self-monitoring, action planning, problem-solving, relapse prevention, social support and feedback. During Week 6 of CR, participants were provided with a validated pedometer and instructed to wear it daily and record step counts and/or PA in activity diaries. Weekly face-to-face reviews with the researcher were conducted for six weeks to support personalised goal-setting (e.g., progressive step-count increases). Following this intensive phase, participants received three monthly follow-up contacts to reinforce behaviour maintenance. Group discussions were facilitated by the researcher during CR sessions to address benefits of PA, barriers and facilitators, social support, and identification of local PA opportunities. The intervention concluded after the 6-month outcome assessment. A full description of the intervention is reported elsewhere [18].

### Nested qualitative study: Sampling and participants

A purposive sample incorporating participants who attended the majority of their intervention arm (>70%) and were able to comment on their experience of the programme were invited to participate in the qualitative study. At their final follow up participants were asked about their willingness to take part in future interviews or focus groups. Those who said they were happy to take part were then invited to do so formally. In the case of one-to-one interviews, a suitable time was agreed between the participant and interviewer. For focus groups, a consensus of preferred days/times was gathered by the researcher and were organised to be held in the slot that would enable best attendance. A variety of methods of data collection were employed (face-to-face, remote video call and telephone) based on the preferences and accessibility of the participants. Meeting details were circulated to those who expressed willingness to participate and those that could make it, did.

### Topic guide

The topic guide, which was developed to address the research aims and iteratively refined throughout data collection. The full topic guide is provided in S1 File. Questions explored contextual influences, including the role of the physical and social environment and personal circumstances, and how these factors affected implementation. The semi-structured interviews and focus groups also included questions relating to participants’ perceptions of the implementation of specific intervention components. Mechanisms of impact (i.e., how the intervention produced change) and perceived effects (i.e., outcomes attributed to the intervention by participants) were also examined.

The interviewer introduced the topics and probed responses, whilst also allowing pauses to encourage reflection and additional insight [28]. An iterative approach was adopted, whereby the topic guide was reviewed and updated after each focus group and/or interview as necessary (NEB and CTMD), in order to better answer the research question and ensure an opportunity to understand the experiences of the participants in greater detail.

### Data collection

Semi-structured interviews were conducted with participants in both trial arms to enable exploration of experiences of maintenance-stage CR and physical activity across conditions, and to allow comparison of contextual influences and perceived outcomes between the control and intervention groups. Interviews were chosen to facilitate in-depth individual accounts, particularly in the control arm where no additional group-based behaviour change sessions were delivered.

In the intervention arm, both interviews and focus groups were conducted. Focus groups were selected to capture shared experiences of the behaviour change programme, explore group dynamics, and examine how collective discussions around goal-setting, barriers and social support may have influenced mechanisms of impact. This approach enabled exploration of both individual perspectives and interactive processes central to the intervention.

A total of seven interviews were conducted with control participants and two with intervention participants. Additionally, two focus groups were conducted with intervention participants (n = 15) (Table 2). The final sample size was determined pragmatically based on participant availability and continued until sufficient depth and diversity of experiences had been obtained to address the study aims.

**Table 2:** Data collection summary.

Method	Intervention Participants (n = 17)	Control Participants (n = 7)
1−1 Interview	2x Interview (1F, 1M) Average Duration: 29m 42s	7x Interview (3F, 4M) Average Duration: 57m 20s
Focus Group (FG)	2x FG (n = 4: 2F, 2M; n = 11: 2F, 9M) Average Duration: 1hr 21m	–

Abbreviations: F = female; M = male.

### Researcher characteristics and reflexivity

The study team included a Principal Investigator (NEB) and co-Principal Investigator (MAT) with extensive experience in the design, delivery and evaluation of cardiac rehabilitation and behaviour change interventions. The project was overseen by both investigators throughout the study. Data collection was led by a researcher (CTMD) who delivered the behaviour change support sessions associated with the intervention but had no prior experience working with this specific population. The cardiac rehabilitation exercise component was delivered by qualified instructors external to the study team.

The researcher’s lack of prior experience with the population may have reduced the influence of pre-existing assumptions during data collection, while oversight from experienced investigators supported methodological rigour and contextual understanding. However, involvement in delivering the intervention may have shaped interactions with participants and interpretation of the data. To minimise potential bias, a semi-structured topic guide was applied consistently, and participants were encouraged to share a range of experiences, including both positive and negative perspectives. Reflexive consideration was maintained throughout data collection and analysis to support balanced interpretation of the findings.

### Analysis

All focus groups and interviews were audio recorded, data were transcribed verbatim by two members of the research team (NEB and CTMD) and validated for accuracy of transcription against the audio files. Anonymised transcripts were imported to NVivo 15 for analysis. The framework method was applied to analyse these data [29]. The analysis was conducted in the following stages: transcription, familiarisation with the interview, coding of the transcripts, developing a working analytical framework, applying the analytical framework, charting data into the framework matrix, and interpreting the data [29]. The initial coding was conducted by two independent researchers (NEB and CTMD). As a next step, a working analytical framework was developed by discussing similarities and differences between codes assigned, until an agreement on a set of codes was achieved. This agreed upon set of unique codes were then used to establish an initial analytical framework, which was then refined by coding of further transcripts by both researchers independently, until no new codes were generated. Once all the data were coded the qualitative research team identified themes, sub-themes and categories (where appropriate) to summarise the main findings within the pre-specified framework matrix.

### Ethical approval

The trial was registered (ClinicalTrials.gov, Registration date: 26-01-2023, ID: NCT05705310) and was approved by the Institute of Nursing and Health Research Governance Filter Committee (Ulster University: RG3_20-1-3.Z). All participants gave written informed consent.

## Results

The findings for the intervention and control groups are reported under the MRC framework of context, implementation, mechanisms of impact and perceived effects. The themes and subthemes relevant to both intervention and control group data are presented in Table 3. The intervention specific findings are presented in Table 4.

**Table 3:** STRENGTH matrix of findings: Common (intervention and control) themes and sub-themes.

Framework	Overarching Theme	Sub-themes
**Context**	Environmental, social and personal factors that influence the maintenance stage CR experience	Social environmental factors
		Physical environmental factors
		Personal circumstances: physical health, pain and comorbidities
**Implementation**	Social and practical enablers identified within the maintenance stage CR experience	Influential role of the trainer
		Peer support and professional oversight as facilitators
		Perception that the class provided a safe space to do supervised activity
**Mechanisms of Impact**	Facilitators that supported positive experience of the maintenance stage CR experience	Reassured by having professional supervision
		Assessment prompted reflection on current health status
		Valued being in a group of people with shared lived experience
**Outcomes**	Perceived benefits of the maintenance stage CR experience	Social aspect of being in the group
		Improvements in mental wellbeing and physical health

**Table 4:** STRENGTH matrix of findings: Intervention specific themes and sub-themes.

Framework	Overarching Theme	Sub-themes
**Implementation**	Barriers and facilitators that can impact motivation to be active	Supportive and tailored care
		External accountability and structure promoted greater adherence
		Frustration around recording own activity accurately (i.e., non-step activity)
		Self-monitoring increased awareness of activity levels – motivated and validated efforts
**Mechanisms of Impact**	Engagement with the behaviour change processes (education, self-monitoring and goal setting)	Increased awareness of the health benefits of PA promoted healthier lifestyle choices,
		Recognition of the importance of being educated in limitations and capabilities
		Perceived benefits of experiential learning which improved exercise confidence
		Sense of accomplishment gained when achieving goals acted as motivator to continue
**Outcomes**	Positive influence on social network	Reduction in concerns regarding the safety of exercise

## Common findings (intervention and control participants)

Findings shared by the control and intervention groups highlight several key factors influencing the implementation and outcomes of the CR programme, offering valuable insights into how external, personal, and structural elements interact to affect participant experiences and results.

### Context

The overarching theme to emerge regarding context was surrounding the environmental, social and personal factors that may influence the maintenance stage CR experience, both positively and negatively. Three sub-themes were identified, including: (i) social environmental factors; (ii) physical environmental factors, and (iii) personal circumstances; physical health, pain and comorbidities. Participants’ ability to engage in and benefit from the intervention was often shaped by these contextual elements, highlighting the importance of understanding the broader environment in which health interventions occur. In terms of social environmental factors, participants reported that their social environment had an impact on their engagement in PA or exercise.

P14 “When I took ill my family just thought, like (P18) said, cotton wool, ‘you can’t do this mum, you can’t do that’, and the husband was saying ‘don’t be lifting that, you can’t clean the windows’.” (Intervention)

Some participants also noted that the physical environment, for example, where they lived (home setting) and proximity to suitable places to do PA and/or exercise had an influence on their ability to be active.

P18 “I don’t really have the space to do the physical exercise, so the gym is really important for me, you know if I wasn’t going to that I wouldn’t be doing that sort of thing.” (Intervention)

In addition to that, participants reported that personal circumstances including their physical health, pain and comorbidities could be a barrier to engagement in PA.

P2 “I have underlying conditions, with multiple morbidities, at a certain age, so yeah… there are some things that hinder me doing things.” (Control)

### Implementation

The overarching theme to emerge regarding implementation was social and practical enablers identified within the maintenance stage CR experience. Three sub-themes were identified, including: (i) influential role of the trainer; (ii) peer support and professional oversight as facilitators, and (iii) perception that the class provided a safe space to do supervised activity. The structures, resources, and procedures used to deliver the programme played a crucial role in its effectiveness.

The trainer’s influence was key to participant engagement in the sessions, and they reported on the positive impact of having a professional supervise the classes.

P3 “Well eh if I’m honest with you, the instructors fantastic. He makes you want to come back because he’s very, he’s very good, he’s very, he’s top, he’s brilliant at what he does.” (Control)

Peer support was also identified as key facilitator of PA.

P17 “Yeah, the social aspect no is, is quite good, […] we’re working as a team now as well you know and supporting each other.” (Intervention)

P22 “The fact that we are together in a group makes a difference, the sense of social for me is very important. So that’s the difference you know.” (Intervention)

Participants reported that the CR class provided a safe space for supervised activity, demonstrating the importance of creating an environment where participants feel supported and secure in their efforts.

P20 “But being able to go somewhere and exercise, knowing that you can push yourself and somebody’s watching is a massive blessing.” (Intervention)

### Mechanisms of impact

The overarching theme that surfaced in relation to mechanisms of impact was the existence of facilitators that supported a positive experience of the maintenance stage CR experience. Three sub-themes were identified, including: (i) reassured by having professional supervision; (ii) assessment prompted reflection on current health status and (iii) valued being in a group with those with shared lived experience. Participants’ responses to the CR maintenance stage programme reflected a combination of psychological and social mechanisms that may have contributed to outcomes. The participants reported that the presence of a fitness professional supervising the sessions offered reassurance.

P9 “It was really good at the start to have a monitor, a heart rate monitor on and they took your blood pressure, so they were able to reassure you very much […] Just someone there to keep saying ‘you’re fine’.” (Intervention)

Participants also reported that the assessments encouraged reflection on participants’ current health, fostering a deeper understanding of their present status and also progress.

P6 “Even down to the questionnaire, know, that you fill in at the start, was good. I found that it mainly asked you everything that you were really thinking, you know, as well. But, its good ‘cause at the end of it you get to see a wee bit of difference in yourself.” (Control)

Additionally, being in a group with others who shared similar experiences created a sense of community and mutual support, further enhancing the perceived value of the programme.

P7 “You kind of look at the like other 12-14 people, whatever in the class, and you’re going through the same thing, and you kind of encourage each other to do, to work on it, you know. […] I found that very, very helpful because you are going through, through the same thing and you could have a chat and laugh about it.” (Control)

P11 “It instils confidence in general to get, to get out there really, you know, and it teaches you your limitations, and it is the input from everybody that does that.” (Intervention)

### Outcomes

The overarching theme to arise regarding outcomes concerned the perceived effects of the maintenance stage CR experience. Two sub-themes were identified, including: (i) social aspect of being in a group, and (ii) improvements in mental wellbeing and physical health. Participants valued the social aspect of the group, which extended beyond PA and tangible physical gains, to provide a sense of camaraderie and connection.

P20 “I live on my own, so the classes are where I would meet people, cause otherwise I’ve got no encouragement to do anything. I could just, just be in the house 24/7 and nobody would notice, so the classes are really important for me.” (Intervention)

The participants also reported that the CR programme resulted in several positive outcomes, including perceived improvements in both mental wellbeing and physical health.

P3 “I can feel the benefits you know what I mean, I can feel myself getting that wee bit fitter and stronger in terms of what I was two years ago.” (Control)

P23 “I’m still kind of pinching myself to get to this point a year on where I think I you know I haven’t come across anything that I actually can’t do.” (Intervention)

## Intervention specific findings (intervention participants)

The intervention-specific findings provide insights into how specific elements of the behaviour change intervention contributed to participant engagement, motivation, and overall outcomes.

### Implementation

The overarching theme to emerge with regards to implementation was in reference to barriers and facilitators that can impact motivation to be active. Four sub-themes were identified, including: (i) supportive and tailored care; (ii) external accountability and structure promoted greater adherence; (iii) frustration around recording own activity accurately (i.e., non-step activity), and (iv) self-monitoring increased awareness of activity levels – motivated and validated efforts.

Participants reported that receiving tailored and supportive care from the trainer/researcher played a key role in promoting engagement with the programme.

P24 “No yeah no I think just remembering, getting, hearing from somebody saying ‘how are you doing?’ is important, it reminds you that, well, have I been doing it? Gee I’m glad somebody’s checking, and those kinds of things.” (Intervention)

P12 “To come in and take your blood pressure, check your weight and all that kind of thing, so it’s just, that there is somebody there looking after you […] I think that all those things just gave you a feeling that you’re getting watched […] not even accountability, but somebody, somebody that cared for you, you know.” (Intervention)

Self-monitoring activity levels was also identified as a motivational tool, as it increased awareness of progress and validated participants’ efforts.

P13 “The pedometer I thought was great. I didn’t at first. I didn’t kind of understand where I was going or what I was doing. But as the days and the weeks crept up it was interesting to look at what I had done, because I kept a record, and I still continued on doing that just for my own sake.” (Intervention)

However, some frustration was expressed around accurately recording non-step activities, highlighting challenges in the the self-monitoring process.

P23 “Well the frustration thing with I mean like doing gigs and drumming for example and em, (I’d) be on tour each night, be real high energy and stuff that takes everything out of you and nothing would record it.” (Intervention)

Despite this, participants perceived that external accountability, and the structured nature of the programme were important factors that encouraged greater adherence and consistent participation.

P8 “Well, that they help with the motivation process. It’s like, you know, if they say ‘what goal do you want to set yourself?’, you feel sort of obligated to at least try and meet it.” (Intervention)

### Mechanisms of impact

The overarching theme identified relating to mechanisms of impact was surrounding engagement with the behaviour change processes (education, self-monitoring and goal setting). Four sub-themes were identified, including: (i) increased awareness of the health benefits of PA promoted healthier lifestyle choices; (ii) recognition of the importance of being educated in limitations and capabilities; (iii) perceived benefits of experiential learning, which improved exercise confidence, and (iv) sense of accomplishment gained when achieving goals acted as a motivator to continue.

The intervention increased participants’ awareness of the health benefits associated with PA, which motivated them to improve their health status.

P9 “They (the monitoring tools) did have an influence on my activity levels because I didn’t want to let myself down you know even if it was raining I thought, well, get the rain coat on and go for a walk to get the steps up, which I don’t do now but it was good, it gave me that wee catalyst to keep going.” (Intervention)

P16 “On days when I haven’t been for a walk or I haven’t done, I feel guilty about it, whereas in the past I just wouldn’t have worried about […] whether I’d walked or not.” (Intervention)

The process of education, especially understanding both personal limitations and capabilities, was important for boosting participants’ confidence.

P10 “Initially I didn’t have the confidence, you know, now I try to push myself, within reason.” (Intervention)

Due to the nature of the programme (i.e., cardiac specific), participants were training with individuals with shared life experiences. Participants noted that being in a group with others who had similar conditions or had undergone surgeries gave them an opportunity to ask questions and ease concerns surrounding what they could and could not do. The supervised group-based element also provided an opportunity for experiential learning. This experiential learning allowed individuals to better understand their own bodies, fostering the ability to manage their progress.

P11 “I think that stems originally from the class and that encouragement initially to give you that kick start… that kick up the backside you need” (Intervention)

P24 “It was good to get back and realise that you know I can do this and I can do that without my chest flopping open again, […] you literally have to take the weights and raise them up, and you find out you can do it.” (Intervention)

Achieving set goals that were meaningful to the individual provided a sense of accomplishment, acting as a strong motivator for maintaining activity levels over time.

P13 “If I’ve gone and I’ve done say 6,000 steps today, it’s like, I really did good and its mental health that way in that I get a pat on the back from myself.” (Intervention)

### Outcomes

The positive influence on the participants’ social network emerged as the overarching theme pertaining to outcomes. One sub-themes was identified: reduction in concerns regarding the safety of exercise. Participants reported that beyond individual health improvements, education surrounding the health benefits of exercise and specific cardiac guidelines had a broader impact, extending to their family and friends. Educating family and friends on their high level of safe physical capability relieved a certain amount of concern in their loved ones. These incidental effects demonstrate the far-reaching potential of educational interventions, where benefits can extend beyond the individual to their social network.

P14 “I think when you have something happens in your heart people think you can’t do this, and you can’t do that. It educates you know, and they did teach you that your heart is a muscle, and the tablets will help half the way, but you have to do the other half. If you didn’t go to those classes, you wouldn’t be lifting, you wouldn’t be climbing, you wouldn’t be, you know so it’s an education. It is definitely an education.” (Intervention)

### Adherence to CR programme

The attendance rate during the 12-week maintenance stage of CR was 67% for participants in both the control and intervention groups.

### Fidelity of intervention implementation

Four of the six CR classes involved in the study practiced rolling recruitment, one did not recruit at all but received referrals from other centres after the participants initial onboarding to the CR programme, and only one centre recruited in blocks aligning with the original study design. While individuals in the two non-recruiting/block recruiting classes received the intended communication and support from the researcher, the rolling recruitment in the other four classes required the researcher to be present weekly. This made the researcher more accessible to those attending weekly classes at those centres, beyond what was described in the protocol. This extended access meant that, though researcher oversight should be stepped down to monthly after the sixth week of the intervention, some participants may have greater perceived oversight/support and therefore motivation, due to the weekly presence of the researcher. Indeed, many participants would verbally report their step counts to the researcher whenever the researcher was present, regardless of follow up schedule. Moreover, of the 78 participants that remained in the study until their 6 month follow up, almost half (n = 37; control = 13, intervention = 24) were still at that point attending CR classes at least occasionally.

### Adherence to intervention

On average, participants adhered with the self-monitoring aspect of the intervention 87% of the time, and the goal setting aspect 58% of the time. Self-monitoring adherence declined over time and with decreased researcher oversight (Table 5). Percentages represent the percent of the cohort that recorded their activity or set a goal at each follow up timepoint. Data were classed as missing if the participant remained in the study but was unreachable at that given timepoint, it is therefore unknown whether the participant self-monitored their activity or set goals. Drop-outs refer to participants who had withdrawn from the study at that given timepoint.

**Table 5:** Adherence rates of physical activity (PA) self-monitoring and goal setting across the course of the study.

Intervention contact timepoint	PA self-monitoring adherence		Goal setting adherence	
	(including missing and drop-outs)	(excluding missing and drop-outs)	(including missing and drop-outs)	(excluding missing and drop-outs)
Follow up 1 (weekly)	87%	96%	56%	62%
Follow up 2 (weekly)	85%	96%	50%	57%
Follow up 3 (weekly)	81%	93%	46%	53%
Follow up 4 (weekly)	79%	91%	46%	56%
Follow up 5 (weekly)	75%	87%	52%	60%
Follow up 6 (weekly)	77%	91%	58%	68%
Follow up 7 (monthly)	56%	78%	42%	59%
Follow up 8 (monthly)	52%	69%	37%	49%
Follow up 9 (monthly)	67%	78%	–	–
Average	73%	87%	48%	58%

S1 Fig provides a visual representation of the trajectory of participants step goals versus actual steps achieved over the course of the intervention.

## Discussion

This study is part of the larger STRENGTH study, which used mixed methods to develop, implement and evaluate a complex behaviour change intervention. The qualitative study explores the experiences of individuals who participated in a behaviour change intervention compared to those who received standard care at the maintenance stage of their CR programme. The process evaluation element also evaluates the intervention’s impact on PA adherence and identifies its strengths and limitations from participants’ perspectives to inform future CR programme delivery.

Key contextual factors impacted both the implementation and outcomes of the programme. Many participants reported a perceived lack of confidence in exercise independently or unsupervised, which likely affected their ability to engage fully with the intervention side of the programme outside of structured sessions. Additionally, uncertainty regarding the perceived safety of physical exertion, given existing health conditions, may have further contributed to hesitation in participating in moderate-vigorous exercise. This has been shown in other studies, whereby barriers including concerns about adverse events or safety have been reported [30,31]. These contextual barriers highlight the importance of facilitating a supportive environment for exercise, particularly for those with concerns about safety and ability. A recent cross-sectional survey by Fraser et al. reported that self-efficacy increased as a result of attending CR, partly due to the safe environment it provided [32]. Similarly, a study in Korea found that social support from healthcare professionals directly promotes PA and also has an indirect effect by boosting both self-efficacy and autonomous motivation in individuals with coronary artery disease [33]. Critically, this study recognised the importance of supporting autonomy in the promotion of PA. While our participants valued support from knowledgeable staff, and felt reassured by the expert supervision, participants’ concerns around independent exertion remained. This may have fostered a reliance on professional oversight when exercising. Therefore, future efforts should focus on building confidence in unsupervised exercise to promote independence – an essential step in reducing the burden on professional CR support systems.

Fraser et al. also found that exercising with similar individuals, an inherent feature of participating in the CR programme, increases self-efficacy [32]. Similarly, the STRENGTH study highlighted the importance of the social aspect of CR, with participants valuing the opportunity to connect with others who had shared lived experiences and appreciating peer support, which encouraged participation in group exercise. Interestingly, despite participants reporting the positive impact of the social environment (both professional and peer) on self-efficacy and mental wellbeing in these qualitative analyses, our quantitative findings, including self-efficacy, weekly physical activity levels, and mental wellbeing did not identify any measurable change from baseline to the end of the intervention [18]. In other words, the intervention did not lead to increases in PA, and participants in the intervention arm did not differ significantly from the control arm across any timepoints. Similarly, changes in self-rated PA self-efficacy, quality of life, and mental wellbeing were comparable between arms. The misalignment in our findings suggests that either, our quantitative measures are unable to fully capture the nuance of social benefits, or that these social benefits are present at the core stage of CR and our cohort are not distinguishing between different CR stages in their responses. It is likely a combination of both, and efforts should be made in future studies to refine measurement tools and better understand how social benefits evolve across CR stages, allowing us to disentangle and overcome these confounds. In addition, including measures that evaluate social health aspects such as loneliness, social network and social connectedness would be useful to understand the potential impact on these outcomes. Notably, none of the quantitative measures specifically captured social health dimensions such as peer connectedness or group cohesion, which may explain why social benefits were detected only qualitatively.

While social environment often arises as a mediator of PA in health studies [26,34], the benefits or constraints imposed by the physical environment on PA maintenance is a more novel insight. Physical environments in the current study were found to facilitate accumulation of steps for some (e.g., through house layout, or access to safe and enjoyable walking areas nearby), but to limit activity outside the CR class for others (e.g., through having little space at home for PA, or living in areas perceived as unsafe for walking). As well as the physical structure of spaces impacting PA, mental associations between certain environments and the participants condition (e.g., references to “cardiac hill”, or avoidance of locations of prior cardiac events) were revealed. Future research on PA in healthcare interventions should consider how associations between physical environments and cardiac events, or other health conditions more broadly, might influence outcomes.

The educational component of the overall CR programme, which focused on understanding physical capabilities, was beneficial not only for participants but also for their social circle. Participants often shared their learning with family and friends, promoting broader health benefits and helping to reduce loved ones’ concerns by demonstrating that participants could safely engage in high levels of PA. Some participants also reported that their loved ones felt more at ease knowing they had a space for exercise with professional assistance at hand. These findings align with a qualitative study exploring the experiences of CR participants identified three main elements of CR, including exercise, education and environment [35]. Authors noted the importance of educational classes in providing essential content for a healthy lifestyle and the role of supportive staff in boosting participants’ confidence to resume exercise and determine appropriate levels of engagement.

Adherence rates of participants in this study were in line with adherence rates seen in other PA interventions among those with CVD. A meta-analysis estimated pooled adherence rates for patients in the intervention groups of CVD populations to be 90% (95% Confidence Interval [0.83, 0.96]) [36]. Interestingly, participants were consistently less likely to set a PA goal than to self-monitor their activity, potentially owing to the retroactive nature of self-monitoring versus the proactive nature of goal setting. Indeed, the researcher noticed a considerable reluctance to setting goals in many participants. Consequently, without external prompting, this aspect of the intervention was often bypassed. While there is considerable literature on the impact of goal setting on adherence to behaviour change interventions [37], adherence to independent goal setting has been less well considered.

Overall, our findings highlight the specific mechanisms by which the STRENGTH behaviour change intervention influenced participant engagement and perceptions of PA, including enhanced self-efficacy, structured self-monitoring, and peer support. These insights provide actionable guidance for embedding behaviour change strategies into maintenance-stage CR programmes.

### Strengths and limitations

This evaluation was conducted within the STRENGTH study cluster randomised controlled trial. Our participants were comprised of both men and women, of a broad age range, within both control and intervention groups. The one-to-one interviews were undertaken by an interviewer not previously known to the participant, to encourage greater honesty in responses to questions about participant experience, particularly regarding interactions with the researcher/instructors. The researcher was present at the focus groups but did not lead the discussion. Analysis and interpretation of transcripts were completed by two researchers independently before coming together to identify shared findings.

The self-selection process for participating in interviews/focus groups may limit how much the statements reflect the intervention participants more broadly. A common reason for declining to participate in focus groups was time constraints/work, factors that may also impact availability to meet PA guidelines. Furthermore, the cultural backgrounds of those present in Northern Ireland in general has a high degree of homogeneity. This homogeneity may therefore limit the broader applicability of findings conducted in this population across similar cohorts in other countries.

## Conclusions

Despite the positive feedback regarding the social and educational aspects of the programme, there were no significant changes in the measures of PA outcomes, quality of life, self-efficacy, or mental wellbeing scores between groups or over time [18]. Similarly, no meaningful changes were observed in anthropometrics, blood pressure, or resting heart rate, suggesting that, while participants may have found the programme beneficial in terms of knowledge and social support, it did not lead to measurable improvements in physical health markers. Qualitative findings did, however, highlight a perceived lack of exercise confidence, which may have been a barrier to greater engagement with the independent PA as part of the intervention. Additionally, participants emphasised the importance of education, particularly in understanding their own exercise capacity, as a key factor in fostering confidence and engagement with the intervention.

## Supporting information

S1 File Topic guide. (DOCX)

S1 Fig STRENGTH intervention participants’ step goals versus steps completed across the course of the 6 month intervention. (DOCX)

## Abbreviations

CHD: Coronary Heart Disease
CR: Cardiac Rehabilitation
CVD: Cardiovascular Disease
DoH: Department of Health
MVPA: Moderate-Vigorous Physical Activity
RCT: Randomised Controlled Trial
PA: Physical Activity
SD: Standard Deviation
STRENGTH: Self-management and Theory-based Rehabilitation Encouraging New Gateways to Healthy-Hearts
WHO: World Health Organization
