# Supplementary material for: Understanding participants’ experiences of a behaviour change intervention within cardiac rehabilitation: A nested process evaluation within the STRENGTH randomised controlled trial
Source: PLoS One. 2026 Jun 16;21(6):e0351117. doi: 10.1371/journal.pone.0351117 (PMC13271477; doi:10.1371/journal.pone.0351117)
Supplement: S1 File — (DOCX) [file pone.0351117.s001.docx]

**Study Title:** The STRENGTH Study: Self-management and Theory-based Rehabilitation Encouraging New Gateways to Healthy-Hearts

**Topic Guide**

**Implementation**

What motivated you to agree to take part in this study?

- Was it what you expected?
- In what ways?

What were your general thoughts of the STRENGTH intervention and exercise programme?

Overall, how satisfied were you with the programme (what components did you like/dislike)?

- What specific features of the intervention impressed you? (if any)
- What features of the intervention disappointed you? (if any)

What, if anything did you find frustrating or unappealing about the programme?

Were any of the tasks suggested in the programme difficult for you to perform or maintain once the programme ended?

How likely would you be to recommend this programme to a family member or friend? Why?

What was your experience with the assessments (including the actigraph)?

**Perceived Effects**

Do you think the intervention had any effects (positive or negative)?

- If so, have any lasted?

Have you perceived any specific effects (i.e. general health, mental health, physical health, social relationships)?

During the programme did you increase your physical activity?

- If so, why do you think that you have (confidence, physical capacity, other reasons)?
- If not, why do you think this is so?

Do you think you will maintain your increased PA once the programme has ended?

- If so, in what manner (walking, group-based classes, other)?
- If not, why do you think that?

**Mechanisms of Impact**

How did you find completing the activity diary and monitoring your steps?

Can you tell me about the strategies you have used to accomplish each session’s goals?

What were the reasons behind you accomplishing (or not) the goals?

Did any components of the intervention influence this?

- Raising awareness on differences, associations, risks and benefits of PA.
- Self-monitoring (pedometer and activity diary)
- External monitoring (instructor)
- Raising awareness on facilitators and barriers of PA
- Peer and social support from the group
- Telephone prompts

**Context**

*Physical Environment*

How would you describe the physical environment of where you live?

What did you think of the setting of the intervention (i.e. facilities used for the exercise programme)?

Have you started activities in your local community since the end of the programme?

How do you perceive where you live when considering whether and how certain aspects can affect being physically active?

- Availability of places within walking distance/short drive to do structured exercise
- Parks, leisure centres, other indoor and outdoor activities
- Weather or seasonal changes

*Personal Networks*

Do you think your personal situation or health status helps or hinders your ability to be physically active?

- How and why?

Do you think your personal network (partners, family, friends) helps or hinders your ability to be physically active?

- How and why?

**Concluding Questions**

Do you have any suggestions on how the programme could be improved?

- Type of activities, duration of sessions, frequency of training

Is there anything that we haven’t covered that you would like to add?
