# Supplementary material for: Understanding participants’ experiences of a behaviour change intervention within cardiac rehabilitation: A nested process evaluation within the STRENGTH randomised controlled trial
Source: PLoS One. 2026 Jun 16;21(6):e0351117. doi: 10.1371/journal.pone.0351117 (PMC13271477; doi:10.1371/journal.pone.0351117)
Supplement: S1 Fig — (DOCX) [file pone.0351117.s002.docx]

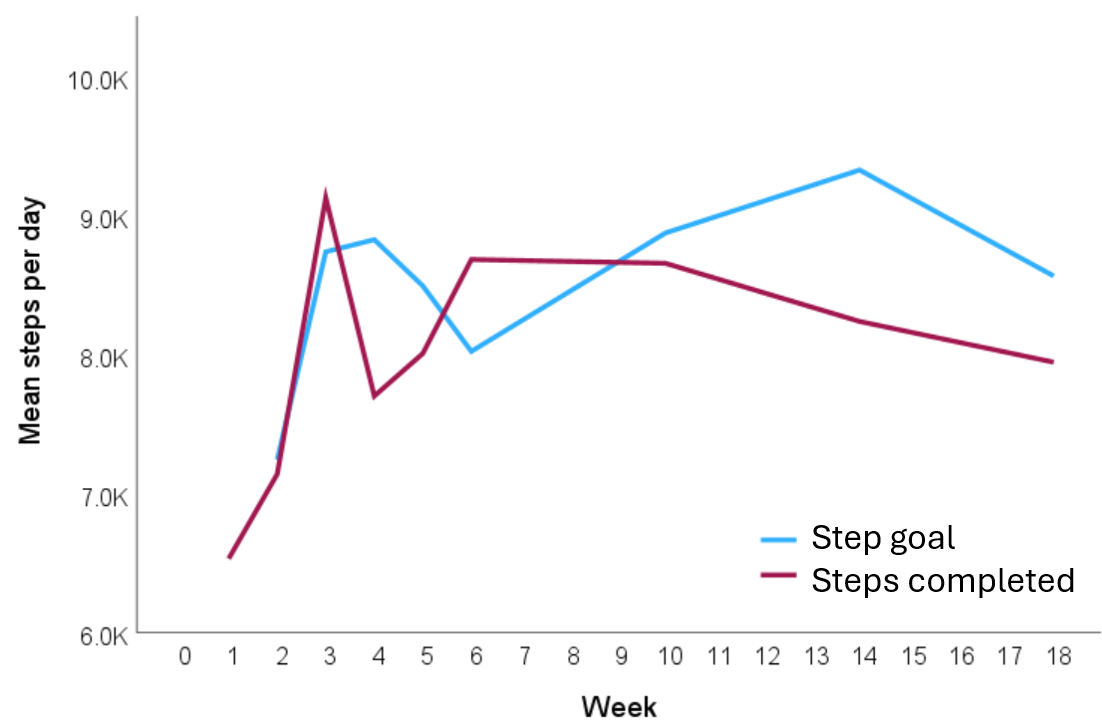


**S1 Fig.** STRENGTH intervention participants’ step goals versus steps completed across the course of the 6 month intervention.
